# Income change and sympathy for right‐wing populist parties in the Netherlands: The role of gender and income inequality within households

**DOI:** 10.1111/1468-4446.13122

**Published:** 2024-07-09

**Authors:** Yoav Roll, Nan Dirk De Graaf

**Affiliations:** ^1^ Nuffield College University of Oxford Oxford UK; ^2^ Department of Sociology University of Oxford Oxford UK

**Keywords:** gender, Netherlands, panel data, right‐wing populism, within household inequality

## Abstract

The global rise of right‐wing populist [RWP] parties presents a major political concern. RWP parties' voters tend to be citizens who have either experienced or fear economic deprivation. Income change constitutes a viable measure of this deprivation. However, previous contributions examining effects of income change on support for RWP parties have yielded diverging conclusions. This paper challenges previous findings by incorporating considerations of gender and within‐household inequality. We hypothesise a negative relationship between, on the one hand, personal and household income change and, on the other hand, sympathy towards RWP parties. Furthermore, we expect to find a stronger association between personal income change and RWP sympathy among men. Moreover, we expect the relationship between household income change and RWP sympathy to differ between genders. Finally, we hypothesise that this gender disparity can be interpreted by considering who contributes most to the household income. All these hypotheses are grounded in gender socialisation and economic dominance theories. Analysing Dutch LISS longitudinal data spanning from 2007 to 2021 (*N* = 7,801, *n* = 43,954) through fixed‐effects multilevel linear regression models enables us to address various competing explanations. It appears that only for men, personal income change is negatively linked with sympathies towards RWP parties. However, considering who is the highest earner within households reveals that women are also affected by their personal income change if they earn the highest income. For both men and women, household income change is negatively linked with sympathies towards RWP parties. These results lend partial support to both the socialisation and economic dominance theories. The implications of these findings are discussed.

## 1 INTRODUCTION

The worldwide rise of right‐wing populism constitutes a major political development. In Europe, right‐wing populist [RWP] parties have transitioned from fringe groups to influential political entities since the late 1990s (e.g., Mudde, 2019). Economic changes, such as globalisation, deindustrialisation, automation, and welfare‐state retrenchment, are often considered to be among the main causes affecting right‐wing populism's emergence (e.g., Colantone & Stanig, 2019; Guriev & Papaioannou, 2022; Rodrik, 2018; Scheiring et al., 2024; Steiner et al., 2024). Some scholars theorise that populism emerges from tensions arising between those who benefit and those who suffer from recent economic shifts (e.g., Steiner et al., 2024). Those adversely affected by these changes, ‘the losers’, are typically lacking academic qualifications, workers in blue‐collar occupations, the self‐employed or unemployed, and those with only low or medium‐low incomes. Conversely, ‘the winners’ tend to be the academically educated, trained professionals, managers, and those earning high incomes (e.g., Arzheimer, 2017; Diermeier, 2020; Hobolt, 2016; Rydgren & Ruth, 2013). Economic grievances among the ‘losers’ may manifest in the form of populist backlashes against those perceived as benefiting from recent changes (e.g., Diermeier, 2020; Hochschild, 2018; Steiner et al., 2024; Wuthnow, 2018).

Income, alongside employment status and occupation, serves as a reliable indicator of a person's economic standing. Consequently, objective income change can serve as a measure of economic deprivation (Gidron & Mijs, 2019; Hartmann et al., 2022). However, previous research investigating the link between objective income change and support for right‐wing populism draws a complicated and contradictory picture, with some studies finding limited evidence for this connection (Gidron & Mijs, 2019) and others finding no evidence thereof (Hartmann et al., 2022). Some scholars have instead turned towards testing the impact of relative and subjective measures of income on RWP support (e.g., Burgoon et al., 2019; Hartmann et al., 2022; Kurer, 2020). However, given the empirical connection observed at the macro‐level between objective economic decline and support for right‐wing populism (e.g., Anelli et al., 2021; Colantone & Stanig, 2019; Guriev & Papaioannou, 2022; Scheiring et al., 2024), it remains imperative to understand this relationship on the micro‐level. Grasping this micro‐to‐macro connection is essential if we are to substantiate any potential causal link between economic change and shifts in RWP support (see Coleman, 1990). Therefore, this paper seeks to challenge and expand upon previous findings by identifying crucial missing pieces in the puzzle that elucidate the impact of income change on RWP support. Gender and within‐household income inequality play a key role in this endeavour.

It is almost a universal finding that women are less likely to support RWP parties. Some scholars attribute this divergence to distinct behaviours between men and women that appear even when they occupy similar economic roles (e.g., Abou‐Chadi & Kurer, 2021; Gidengil et al., 2005). Various socialisation theories may elucidate how gender conditions the connection between economic status and RWP support (e.g., Gidengil, 1995; Oshri et al., 2023; Sipma et al., 2023a). However, they all converge on a shared expectation: women tend to respond more to social context, while men tend to prioritise their own economic situation in determining their political attitudes and behaviour.

We propose an alternative economic explanation based on household income inequality: the persistent male breadwinner role elevates men's income into a more decisive factor in determining economic deprivation and, consequently, political attitudes and behaviour. This phenomenon is particularly notable in the Netherlands, a country which, compared to Western‐Europe, has maintained relatively conservative gender roles within families, where women tend to fulfil most household duties and commonly maintain only part‐time employment (e.g., Esping‐Andersen, 2009).

Thus, we will address three main questions: Firstly, to what extent does income change influence sympathies towards RWP parties? Secondly, does the relationship between income change and RWP sympathy differ between genders? Thirdly, can gender disparities be elucidated by considering who earns most within the household?

Concerning the first question, we hypothesise a negative correlation between income change − both personal and household − and sympathies towards RWP parties, whereby an income decline (increase) leads to sympathetic (unsympathetic) attitudes towards RWP parties. We expect this pattern to hold true for both men and women. Regarding the second question, we hypothesise that compared to women, men's personal income change will exhibit a stronger association with their sympathies towards RWP parties. Furthermore, we predict that, for women, household income will have a greater impact on RWP sympathy than personal income. We note that the socialisation theory leads us to contradicting hypotheses regarding household income change. On the one hand, women, compared to men, are presumed to respond more to social context, which includes the household (and its income). On the other hand, women, compared to men, are also expected to prioritise broader socio‐cultural issues over economic circumstances (including household and personal income). Thus, we hypothesise a gender disparity in the relationship between household income change and RWP sympathy. Finally, regarding the third question, we hypothesise that a change in personal income for the highest earner in the household correlates with a change in RWP sympathy for the same partner.

We test our hypotheses by focusing on the Netherlands. The Dutch context, combining a pluralistic and representative multi‐party system with a robust economic and cultural interconnectedness, serves as an exemplar of the surge of right‐wing populism in Western Europe. To test our hypotheses, we implement fixed‐effects multilevel linear regression models, with fixed‐effects for years, on panel data collected between 2007 and 2021, constituting 14 waves of the Politics and Values module (Das & Elshout, 2018; Elshout & Centerdata, 2022) of the Longitudinal Internet studies for the Social Sciences [LISS] panel, as administered by CentERdata at Tilburg University (see Scherpenzeel & Das, 2018). Our design enables us to control for all time‐invariant differences among individuals, and any contextual social, economic, and political change occurring in the Netherlands during the time span, including, for instance, any increase in the number of immigrants and economic downturns. Thus, many alternative explanations are already accounted for.

We find no evidence of a connection between personal income change and attitudes towards RWP parties when disregarding gender. However, in line with our hypothesis, when considering gender, we observe a negative correlation between personal income change and sympathy towards RWP parties *among men only*. Furthermore, as predicted, we identify a negative association between household income change and sympathy towards RWP parties for both men and women. In particular, we find that, for women, the impact of personal income on RWP sympathy is significantly lower than that of household income. However, among women who outearn their partners, we demonstrate a negative correlation between personal income change and RWP sympathy, much like that of men when they are the main household earners. Finally, separate analyses of income decline and increase yield consistent outcomes. The results of this paper give support to both the socialisation and the economic dominance theories.

## 2 RIGHT‐WING POPULISM AND THE DUTCH CONTEXT

Populism is commonly understood as an ideology that divides society into two opposing and antagonistic groups: ‘the corrupt elite’ versus ‘the pure people’. In the populist imagination, the people are seen as hard‐working, honest, and virtuous citizens, endowed with common sense, while the elite is seen as unscrupulous, detached from the people's concerns, and conspiring with foreign elements (Mudde, 2007, p. 23, 2019). Right‐wing populism amalgamates populism with authoritarianism and nativism (Mudde, 2007, 2019). Nativism may be defined as xenophobic nationalism, viewing immigrants and members of ethnic minorities as a danger to the homogenous nation‐state. Authoritarianism is often understood as a disposition for stricter social control and severe punishments to those who deviate from social norms and laws (Mudde, 2007, 2019).

To better understand where RWP parties position themselves in the wider political spectrum, one may consider Hooghe et al.’s (2002) innovative proposition of a dichotomous axis of distinction between political parties in Europe, running perpendicularly to the old economic left to right spectrum. The result is a clear delineation between, on the one hand, traditional/authoritarian/nationalist (TAN) parties, to which they sort RWP parties, and on the other hand, green/alternative/libertarian (GAL) parties, both groups representing starkly opposing attitudes on a variety of novel policy issues, such as immigration, European integration, and the environment. Similarly, Norris and Inglehart (2019) regard right‐wing populism as a reaction against post‐materialist politics.

In the Netherlands, the main RWP party today is the Party of Freedom [PVV] (Oudenampsen, 2020; Vossen, 2011, 2017). Established in 2006, the party initially garnered 6.9% of the vote that year, subsequently surging to peak support of 23.5% in the 2023 elections. The Dutch electoral system, featuring a blend of proportional representation and an exceptionally low entry threshold, provides fertile ground for the birth of new parties (Vossen, 2017), while facilitating a close match between party supply and demand. Consequently, other populist parties emerged in the wake of the PVV's success, such as the failed Proud of the Netherlands Party [TON], the Forum for Democracy [FvD], and its offshoot, Right Answer 2021 [JA21]. Collectively, the combined vote share for RWP parties in the Dutch parliament peaked at 26.4% during the 2023 parliamentary elections.

Historically renowned for its multiculturalism, the Netherlands boasts a reputation for tolerance and liberalism (Entzinger, 2003). At the same time, it is also commonly considered as conservative regarding the gendered division of labour (e.g., Esping‐Andersen, 2009, 2016; Mandel, 2009). Compared to the average Western European women, married Dutch women are more likely to be employed in part‐time work, shoulder a larger share of household responsibilities, and earn lower salaries than their partners (Esping‐Andersen, 2016). Nonetheless, the Netherlands also shares similarities with gender‐egalitarian Scandinavian countries, boasting high female labour force participation rates, particularly among mothers (Knight & Brinton, 2017).

Dutch RWP parties then cater to a combination of anti‐immigration and in particular anti‐Islam sentiments, mixed with certain socially liberal stances. Concepts such as ‘Homonationalism’ and ‘Femonationalism’ (Farris, 2017; Spierings, 2021; Verloo, 2018), have been introduced to characterise how Dutch and other Western European RWP parties' combine favourable positions on sexual and gender identity issues with nativism and authoritarianism. In the Netherlands, RWP parties often present themselves as the champions of women's, gay and lesbian rights against perceived threats from immigrants and Islam (Fiers & Muis, 2021; Lancaster, 2020; Mudde & Kaltwasser, 2015; Spierings, 2020, 2021). However, as noted by Verloo (2018), Dutch right‐wing populism often frames feminism and gender as entirely revolving around their anti‐Muslim agenda, while denying any other feminist and gender concerns.

The PVV manifesto, in particular, revolves around anti‐immigration rhetoric and Islamophobic conspiracy theories, along with anti‐elite sentiments, Dutch nationalism, welfare chauvinism, and authoritarian ideals. The party sees itself as representing the interests of the archetypal Dutch everyman couple, ‘Henk and Ingrid’, who are the only well‐deserved recipients of state welfare and protection (Vossen, 2017). This ideological blend of right‐wing to far‐right cultural attitudes with centre to even centre‐left economic policies, underscored by welfare chauvinism, characterises both the manifesto and political action of Dutch right‐wing populism and the PVV in particular (Verloo, 2018; Vossen, 2017). While RWP parties in the Netherlands tend to draw more support from the working class, less educated, and lower income segments of society, distinctions exist among their respective electorates. FvD and JA21, for instance, tend to attract a more educated base compared to the PVV, although no significant differences are observed in terms of income or occupation among their supporters (Lubbers, 2022). Given the minimal disparities between these parties and the strong correlation in their voters' attitudes, this paper treats all Dutch RWP parties collectively.

Support for right‐wing populism stems from a combination of economic and cultural factors. This paper, as discussed below, focuses on economic factors, while accounting for gender as a sociocultural determinant.

## 3 ECONOMIC THEORIES OF RIGHT‐WING POPULISM AND FINDINGS

Economic shifts have long been recognised as a primary catalyst for the surge of right‐wing populism since the 1980s (Colantone & Stanig, 2019; Funke et al., 2020; Guriev & Papaioannou, 2022; Scheiring et al., 2024). Halikiopoulou and Vlandas (2020) suggest that while the core supporter of the populist right is primarily driven by cultural grievances and nativism, a larger contingent of peripheral supporters backs the populist right due to economic concerns. Although this peripheral group is less likely to support right‐wing populism compared to the core group, its sheer size accounts for a larger part of the total RWP support base.

This economic factor in the support for RWP parties is often intertwined with the fortunes of winners and losers in recent economic trends such as globalisation, deindustrialisation, automation and welfare‐state retrenchment (e.g., Abou‐Chadi & Kurer, 2021; Algan et al., 2017; Autor et al., 2020; Colantone & Stanig, 2018a, 2018b; Frey et al., 2018; Guriev & Papaioannou, 2022; Hochschild, 2018; Rodrik, 2018; Scheiring et al., 2024; Steiner et al., 2024; Wuthnow, 2018). Economic grievances often fuel a backlash against perceived culprits, coupled with welfare chauvinism aimed at preserving social benefits for the perceived in‐group (e.g., Diermeier, 2020; Guriev & Papaioannou, 2022; Hochschild, 2018; Sipma & Berning, 2021; Wuthnow, 2018).

The population most vulnerable to recent economic shifts is largely represented by workers in blue‐collar occupations, by those lacking academic credentials, by the self‐employed and the unemployed. Conversely, the academically educated and those who work in professional occupations tend to reap the benefits of these economic transformations. Correspondingly, research shows that RWP voters mostly belong to the working class or lower‐middle class, the self‐employed, or the unemployed (Abou‐Chadi et al., 2022; Arzheimer, 2017; Autor et al., 2013; Rodrik, 2018; Rydgren & Ruth, 2013; Sipma & Berning, 2021). Anelli et al. (2021) demonstrated that workers in occupations exposed to automation tend to support the populist‐right in Europe. Abou‐Chadi and Kurer (2021) correspondingly found heightened support for RWP parties among individuals whose occupational peers face a higher risk of unemployment. Similarly, in the United Kingdom, Brexit voters were more likely to earn low incomes, lack academic qualifications, experience unemployment, or reside in areas historically dependent on industry (Becker et al., 2017; Goodwin & Heath, 2016; Hobolt, 2016).

With all that said, scholars widely concur that lower and lower‐middle income levels are strong predictors of RWP support (e.g., Antonucci et al., 2017; Engler & Weisstanner, 2021; Hartmann et al., 2022; Hobolt, 2016). Income, alongside employment status and occupation, serves as a reliable indicator of an individual's economic standing. Recent economic changes have affected income in numerous ways, generating winners and losers (Autor et al., 2014, 2019). Some studies indicate that individuals are more inclined to support right‐wing populism when they perceive a shrinking in the gap between their income and that of those at the bottom of the income distribution (Burgoon et al., 2019). Others find that anxiety over loss of livelihood, is more likely to propel individuals towards supporting the populist right (Abou‐Chadi & Kurer, 2021; Kurer, 2020; Sipma et al., 2023b).

To our knowledge, only Gidron and Mijs (2019) have examined the impact of objective income change on support for right‐wing populism using panel data. Utilising the Dutch LISS longitudinal data, which documents both income and political attitudes and behaviour through time, they investigate whether personal income change, measured in Euro, predicts sympathies for the PVV. They found a marginally significant effect of income change on RWP support for those at the top third of income. Hartmann et al. (2022), pairing longitudinal income data together with data on most recent RWP support, studied the effect of household income change, for each quantile of income separately, on people's support for the German RWP party Alternative for Germany [AfD]. While they found that lower quintiles are more likely to support AfD, they also uncovered contradictory patterns when testing for the effects of objective income change: among the lowest two income quintiles, those that had experienced a downward or unstable household income trajectory over the last 10 years, were less likely to vote for AfD, compared to those whose household income either increased or remained stable. No income change effects were found for higher income quintiles.

The findings of Gidron and Mijs (2019) and Hartmann et al. (2022) then present a complex and contradictory picture. While Gidron and Mijs (2019) point to a negative effect mostly among the upper third of income earners, Hartmann et al. (2022) suggest a positive relationship between income change and support for right‐wing populism among lower income groups. As a result, some scholars have abandoned attempts to pinpoint the effects of objective income change on RWP support, shifting their attention to relative and subjective measures of income instead (Burgoon et al., 2019; Kurer, 2020; Sipma et al., 2023b). However, it remains crucial to bridge the gap in understanding between, on the one hand, the well‐documented connection between objective economic decline and rise of right‐wing populism on the macro‐level (e.g., Algan et al., 2017; Anelli et al., 2021; Colantone & Stanig, 2019; Guriev & Papaioannou, 2022) and, on the other hand, the theoretical link between objective income decline and right‐wing populism on the micro‐level. Establishing this link between micro and macro levels is essential to substantiate the association between economic change and shifts in RWP support at the macro level. Without elucidating the micro‐level mechanisms, a potential causality between the two macro‐level phenomena cannot be asserted (Coleman, 1990).

We therefore suggest a new research design testing the relationship between objective decline and support for right‐wing populism, aiming to overcome the disadvantages of previous papers. Firstly, it is evident that income decline is not a linear function; a loss of 1000 Euro for someone earning 2000 Euros monthly differs significantly from a loss of 1000 Euro for someone earning 10,000 Euros monthly. Hence, we assert that a natural logarithm [log] transformation should be applied. Secondly, the household context should not be neglected in this analysis. As discussed below, political behaviour is strongly influenced by household dynamics, including gender and within‐household inequality (Abou‐Chadi & Kurer, 2021; De Graaf & Heath, 1992; Hartmann et al., 2022; Strøm, 2014).

In conclusion, the economic theory of right‐wing populism leads us to expect that income changes serve as the mechanism linking macro‐level economic shifts and support for right‐wing populism. This leads us to the following hypotheses:

**H1a/b.** Personal income change (H1a) and household income change (H1b) are negatively related to sympathy for RWP parties.

We assume (and test) that both an increase in income is related to a decline in RWP sympathy, and a decrease in income is related to an increase in RWP sympathy. Furthermore, we aim to advance theoretical understanding by incorporating a crucial yet overlooked factor in the relationship between economic status and political behaviour: gender.

## 4 THEORIES OF RIGHT‐WING POPULISM AND GENDER

According to Mudde and Kaltwasser (2015), populist parties tend to adhere to the prevailing gender norms in their respective countries. By comparing the gender ideologies of right‐wing populism in Western Europe and left‐wing populism in South America, Mudde and Kaltwasser reveal that Western European RWP parties are, in fact, more progressive compared to South American left‐wing populists. They attribute this disparity to the varying gender norms across different nations.

As previously expounded, Western European right‐wing populists often view immigrants as a threat to women's rights (Farris, 2017; Fiers & Muis, 2021; Lancaster, 2020; Mudde & Kaltwasser, 2015; Off, 2023; Spierings, 2020, 2021). Dutch right‐wing populism in particular embraces many gender‐egalitarian values prevalent in Dutch society, including women's labour force participation, as well as gay and lesbian rights (except for the FvD, which has become reactionary on gender, and politically marginal in recent years). While espousing these values as fundamental Dutch principles, RWP parties focus on the ensuing conflict they perceive with the values brought along with immigration (de Lange & Mügge, 2015; Farris, 2017; Spierings, 2020, 2021).

However, despite RWP parties' general conformity to consensus gender norms, the gender gap in RWP support − wherein men support right‐wing populism more than women − is an almost universal finding (e.g., Akkerman, 2015; Allen & Goodman, 2021; Erzeel & Rashkova, 2017; Mudde, 2019; Mudde & Kaltwasser, 2015; Stockemer et al., 2018; however, see Mayer, 2015). This also holds true in the Netherlands, where, as previously discussed, RWP parties espouse relatively egalitarian stances towards women (e.g., de Lange & Mügge, 2015).^1^ This finding may surprise, given that women typically favour parties that align with their policy interests (Chueri & Damerow, 2023; but see Verloo, 2018, who contends that this is merely superficially the case). The question as to why (and when) this gap emerges has proven to be the focal point of debates in the literature.

Harteveld et al. (2015) notes that for us to understand the RWP gender gap in any context, two components of this gap should be distinguished. Firstly, there are disparities between men and women in measurable attributes, such as income or education, which may lead to divergent levels of support for RWP parties. Put differently, a predictor, such as income or education, mediates the relationship between gender and RWP support. Secondly, the gap is influenced by unmeasured characteristics: differences in how genders behave (politically), despite possessing the same measurable attributes. In other words, gender moderates the relationship between RWP support on the one hand, and predictors, such as income or education on the other hand. The gender gap emerges as a result of both mediating and moderating mechanisms. Our examination of the role of income change in the support for right‐wing populism delves into how gender conditions the relationship between income change and RWP support.

A growing body of research focuses on the differential responses of men and women to their economic circumstances (e.g., Abou‐Chadi & Kurer, 2021; Allen & Goodman, 2021; Coffé, 2013; Fontana et al., 2006; Gidengil et al., 2005; Harteveld et al., 2015; Sipma et al., 2023a). Moreover, prior research suggests that women's political behaviour is determined less by their *personal* economic standing, compared to men, and just as much, if not more, by their household context and by the wider sociocultural context and values (Abou‐Chadi & Kurer, 2021; Chaney et al., 1998; Coffé, 2013; Donovan, 2023; Gidengil, 1995; Gidengil et al., 2005; Sipma et al., 2023a; Welch & Hibbing, 1992).

Some scholars posit that these disparities may stem from gender distinctions in socialisation, which manifest in various ways (e.g., Coffé, 2013; Gidengil, 1995; Gidengil et al., 2005; Welch & Hibbing, 1992). Coffé (2018), for instance, found that, in general, masculine, not feminine personality traits (as determined using Bem's Sex Role Inventory) predict higher support for right‐wing populism. Furthermore, women exhibit a stronger aversion to political parties and movements perceived as stigmatised and/or radical (Harteveld et al., 2019; Inglehart & Norris, 2003). Moreover, although women may demonstrate levels of authoritarianism and nativism comparable to or even surpassing those of men, they are still less likely to support right‐wing populism (Gidengil et al., 2005; Harteveld et al., 2015; Spierings & Zaslove, 2017). In some instances, women may harbour more negative sentiments towards immigrants yet remain less supportive of right‐wing populism (Gidengil et al., 2005; Givens, 2004; however, see Semyonov et al., 2006).

Focusing on how economic circumstances affect political behaviour, Gidengil (1995; Gidengil et al., 2005) suggests a theory of the ‘economic man’ and the ‘social woman’, whereby, based on Gilligan's feminist difference theory, women exhibit more collectivist inclinations, an ‘ethic of care’ (Gilligan, 1982, p. 73), while men display more individualistic tendencies (see also Welch & Hibbing, 1992). Moreover, as Inglehart and Norris (2003) claim, men's political behaviour is more likely to be driven by materialist values (e.g., material wealth), whereas women are more likely to be motivated by post‐materialist values (e.g., environmentalism, self‐expression). Given that right‐wing populism is often seen as the antithesis to post‐materialist politics, this may also explain why it is less attractive for women (Inglehart & Norris, 2016).

The theories discussed above suggest that men are more likely to prioritise their personal economic standing, whereas women tend to focus on their broader sociocultural context and environment when determining their political behaviour. Empirical evidence lends support to these assertions (Coffé, 2013; Gidengil, 1995; Gidengil et al., 2005; Welch & Hibbing, 1992). For instance, Coffé (2013) found that men, compared to women, are more affected by personal economic grievances in their support for RWP. Gidengil (1995) similarly demonstrated that men tend to base their political choices on personal economic considerations, while women tend to place greater emphasis on social objectives, such as welfare and equality. Indeed, Gidengil et al. (2005) demonstrated that household income, and level of education affect men's but not women's support for the populist right in Canada. Correspondingly, Welch and Hibbing (1992) showed that women's political behaviour is less likely to be motivated by their own economic situation, compared with men's. Other studies indicate that while women may be more attuned to context in their political behaviour, this context may also be the economic standing of their household or their partner (e.g., Abou‐Chadi & Kurer, 2021; De Graaf & Heath, 2023; Sipma et al., 2023a; Strøm, 2014).

As discussed above, various socialisation theories offer similar and slightly diverging explanations as to why men and women might respond differently to changes in their economic circumstances. Regarding personal income, all theories point to the same hypotheses: While women may be affected by personal economic considerations, this factor does not influence their RWP sympathies as strongly as it does for men. Moreover, women's personal income is expected to predict RWP sympathy less than household income. Regarding household income, socialisation theories yield two contradicting expectations. On the one hand, we expect that household income may be less strongly associated with RWP sympathy for women compared with men, given women's prioritising of sociocultural context (‘social woman’) rather than pocket‐book economics (‘economic man’; e.g., Gidengil, 1995) in their political decisions. On the other hand, the link between household income and RWP sympathy may also be *stronger* for women than for men due to women's focus on their social context, which includes their household. Consequently, we propose to test the following three hypotheses:

**H2a.** The link between personal income change and sympathies towards right‐wing populism is weaker for women than for men.

**H2b1/2.** The link between household income change and sympathies towards right‐wing populism is (H2b1) weaker / (H2b2) stronger for women than for men.

**H2c.** For women, the link between personal income change and sympathies towards right‐wing populism is weaker than the link between household income change and sympathies towards right‐wing populism.

### 4.1 Income change and income inequality within households

Since socialisation theories yield similar predictions, we are unable to test which of the possible mechanisms is responsible for the gender difference in the link between personal income change and RWP sympathy. However, an alternative theory focuses on women's economic position within the household. As previously outlined, the Netherlands is commonly considered as a conservative country regarding the gendered division of labour (e.g., Esping‐Andersen, 2009, 2016; Mandel, 2009). Compared to the average Western European woman, married Dutch women are more likely to be employed in part‐time work, assume a larger share of household responsibilities, and earn lower salaries than their partners (Esping‐Andersen, 2016). Nonetheless, the Netherlands also shares similarities with the gender‐egalitarian Scandinavian countries in terms of high female labour force participation, particularly among mothers (Knight & Brinton, 2017).

Scholars have proposed four models of understanding how intra‐household social and economic dynamics manifest in the political behaviour of household members: the male dominance model, the economic or class dominance model, the sharing model, and the individual model (see De Graaf & Heath, 1992, 2023). The male dominance model posits that both partners adjust their political behaviour to the economic situation of the man, thus reflecting gendered socialisation where a general dominance of husbands over their wives is assumed. Conversely, the economic dominance model suggests that both partners adjust to whoever has the highest economic status, irrespective of their gender. The sharing model in turn views both partners' economic activity as contributing jointly to the household's political position, such that both partners' economic situations are equally important. Finally, the individual model views each partner in the household as an independent economic and political unit such that their political behaviour is driven by their own economic position.

While empirical evidence indicates a historic trend by which women are decreasingly dominated by the economic standing of their partner in their political party identification (De Graaf & Heath, 2023), recent research still shows that women's political behaviour is partially motivated by their partner's economic standing (Abou‐Chadi & Kurer, 2021; De Graaf & Heath, 2023; Sipma et al., 2023a; Strøm, 2014). As discussed above, Abou‐Chadi and Kurer (2021) explored the impact of occupational risk (the risk of being unemployed in one's occupation) finding that the higher the risk, for both men and women, the more likely they are to support right‐wing populism. Significantly, however, they also found that men tend to weigh their personal economic circumstances more heavily when determining their support for right‐wing populism, whereas women tend to prioritise their partner's economic situation to a greater extent when forming their political attitudes. Nevertheless, Strøm (2014) showed that women vote more independently when they outearn their husband or work full time.

In the Netherlands, the prevalence of the male breadwinner model, coupled with the ubiquity of women's part‐time employment, underscores the importance of acknowledging structural inequalities within Dutch households. A decline in men's personal income consequently tends to exert a greater impact on the overall income of the household. Thus, it is plausible that Dutch women's economic dependence on the family unit may lead them to prioritise their partner's personal income over their own personal income in determining their support for right‐wing populism. Given the persistence of the male breadwinner model in the Netherlands, the economic dominance model may be at play, offering an alternative explanation to socialisation (as represented by the male dominance model) for the gender disparity hypothesised in H2a.

Therefore, considering the Dutch context and previous research, we test whether the economic dominance model applies, leading to hypothesis 3:

**H3.** The personal income change of the partner whose income contributes most to the household income is negatively related to RWP sympathy.

Hypothesis three implies an interaction effect of income on the highest income earner (a dummy variable), potentially interpreting the gender disparity as hypothesised in H2a. Since this theory revolves around interactions between men and women within households, we test hypothesis three on heterosexual couples exclusively.

## 5 DATA AND METHODS

We employ data from the Dutch LISS (Longitudinal Internet studies for the Social Sciences) panel, administered by CentERdata (Tilburg University). LISS is a true probability sample of households drawn from the Dutch population register. It is an online survey, with households that could not otherwise participate being provided with a computer and Internet connection. The LISS panel study began in 2007 and contains multiple topical core modules that are repeated yearly, along with a shorter background monthly catch‐up module, containing socio‐economic and demographic questions (Scherpenzeel & Das, 2018). For this study, the Politics and Values module is utilised (Elshout & Centerdata, 2022), using all currently available 14 waves, collected from 2007 to2021.^1^ Data were selected to include only working‐age respondents (25–64 inclusive).

Data on political variables were collected mostly from December of each year to the beginning of March of the subsequent year. We employ background variables, which were all sampled before the dependent variable, between January and December of each year. For example, if political dependent variables were collected between December 2021 to March 2022, then all independent variables were collected between January to December of 2021. Personal income and household income, our main independent variables, were calculated as the yearly mean of each month's income data, from January to December. Personal and household income data are the net income, with missing values imputed by LISS (see Elshout, 2022). All other independent variables, our controls, were collected from December of each year, if available. If data is unavailable, then it was collected in the previous month of the year, with January of each year being the limit.

The dependent variable is the combined attitudes towards party and leader, ranging from 0 (very unsympathetic) to 10 (very sympathetic) of the four main Dutch RWP parties in the period covered by the study: TON, PVV, FvD and JA21.^3^ There are two reasons behind our decision to utilise attitudes in the analysis, compared with voting behaviour. Firstly, voting depends on many other factors besides political attitudes. For instance, a person may prefer another party for tactical reasons. Alternatively, one may have positive attitudes towards a RWP party and another party at the same time, while choosing to vote for the other party. While one can hold sympathy for multiple parties, one can only vote for one. As we have seen in the recent Dutch 2023 parliamentary elections, sympathy for a party may quickly shift to voting behaviour when political conditions are ripe. Secondly, utilising attitudes data, which is collected annually, instead of every few years, supplies more time points and allows for a more powerful analysis of the longitudinal trend. This is consistent with the work of Gidron and Mijs (2019), which also utilised attitudes from both non‐election and election years.

**FIGURE 1:**
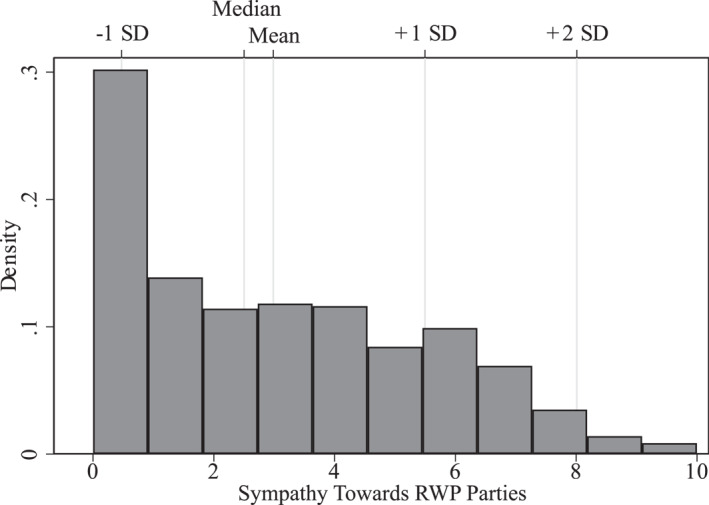
Histogram of sympathies towards right‐wing populist parties in our subsample.

Figure 1 shows the distribution of sympathies towards RWP parties in our sub‐sample. We observe a left‐leaning distribution (median 2.5, mean = 2.98, SD = 2.51), whereby most observations show extremely negative attitudes, with the most common response being 0 (very unsympathetic). The variable was standardised on our subsample.

The independent variables are personal and household net income, corrected for inflation and transformed using the natural logarithm, as well as standardised on our subsample. The use of natural logarithm transformation allowed us to consider that the value of money increases by ratio, not addition. The control variables included in this analysis are those common in sociology of labour: gender, age, level of formal education, unemployment status (if unemployment was experienced at least once in the year), number of children, co‐habitation with a partner, and population density in the area of domicile. The use of longitudinal data, with dependent variables collected after independent variables; control for all time‐invariant variables; and multiple controls for time‐variant variables, which accounts for many alternative explanations; brought us as close as possible to establishing causality within the confines of an observational study. Table 1 includes a comprehensive overview of all variables used.

**TABLE 1:** Variable definitions, percentages, means and standard deviations (*N* = 7780; *n* = 43,954).

Variable	Definition	Mean/percent	SD
Dependent Variables			
Attitudes towards RWP parties	Measured on an 11‐point scale (0 = very unsympathetic; 10 = very sympathetic). Measured between Dec current year and Mar the following year.	2.98	2.51
	What do you think of the party of freedom (Wilder's group)?	2.90	2.75
	What do you think of Geert Wilders?	2.93	2.74
	What do you think of Trots op Nederland (groep Verdonk)? (Verdonk's Dutch pride party) (until wave 3)	3.10	2.56
	What do you think of Rita Verdonk? (until wave 3)	3.48	2.64
	What do you think of the forum for democracy? (From wave 10)	2.64	2.61
	What do you think of Thierry Baudet? (From wave 10)	2.50	2.61
	What do you think of JA21 (Right Answer 2021)? (From wave 14)	3.34	2.62
	What do you think of Joost Eerdmans? (From wave 14)	3.80	2.46
Vote for RWP	Voting for PVV, FvD, JA21, PFL, or 1NL	9.46%	
	2006	5.56%	
	2010	12.94%	
	2012	8.75%	
	2017	11.62%	
	2021	14.37%	
Independent Variables			
Gender	Man = 0	46.10%	
	Woman = 1	53.90%	
Women earn more	For non‐single‐sex couples who are married or co‐habiting:		
	Women earning less than her partner = 0	86.47%	
	Women earning more than her partner = 1	13.53%	
Net income	Average net income of the year (Jan to Dec) at or before at measurement of political attitudes		
	Personal—Income in Euro, corrected for inflation (in 2015 prices).	€1599.23	€2869.77
	Personal (log transformed)—Income in Euro was transformed using the natural log. Those with income lower than 1 Euro = 0.	7.38	1.84
	Household—Income in Euro, corrected for inflation (in 2015 prices).	€2999.53	€4195.88
	Household (log transformed)—Income in Euro was transformed using the natural log. Those with income lower than 1 Euro = 0.	8.01	0.76
Unemployment	Has experienced unemployment at least once in the survey wave = 1	5.97%	
Education	Highest education attained:		
	Low education (primary or VMBO)	23.62%	
	Vocational secondary (MBO)	27.23%	
	General secondary (HAVO/VWO)	8.61%	
	University (HBO or WO)	40.53%	
Age	In years (only working age population [25–64] included)	46.98	11.12
Children	Number of own children in the household	0.92	1.13
Partner	No partner = 0	25.34%	
	Has partner = 1	74.66%	
Domicile	Population density in area of domicile:		
	2500 or more	15.41%	
	1500–2500	25.52%	
	1000–1500	21.85%	
	500–1000	20.45%	
	Less than 500	16.78%	
Year	The year in which the survey was conducted:		
	2007	8.14%	
	2008	8.67%	
	2009	8.51%	
	2010	7.31%	
	2011	7.65%	
	2012	7.30%	
	2013	6.86%	
	2015	7.08%	
	2016	6.57%	
	2017	6.84%	
	2018	6.22%	
	2019	6.31%	
	2020	6.71%	
	2021	5.84%	

*Note*: The individual descriptives are the mean/percentages of the means of individuals through all time points in the sample.

**FIGURE A1:**
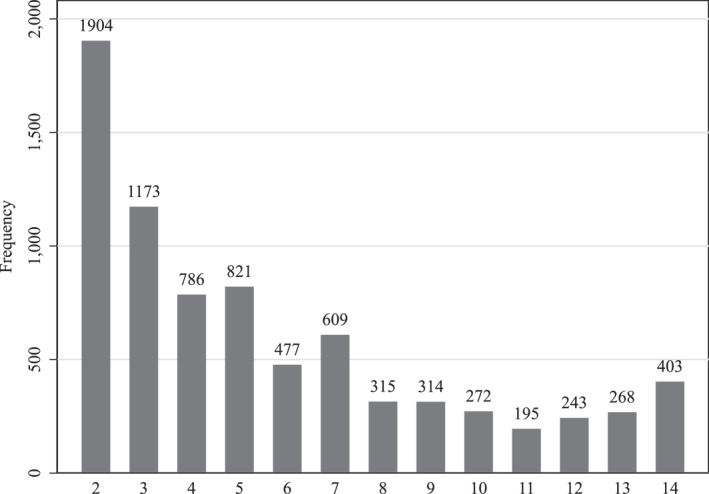
The number of respondents by the number of repeated measures [*N* = 7780].

Our sample includes only observations with data regarding sympathies towards RWP parties. Furthermore, the sample includes only full observations; that is, with no missing values in all independent variables (a loss of 6% of observations). In addition, the sample contains only respondents with at least two observations, as our focus in this paper is to examine within‐individuals change. In total, the sample used in this study contains 7780 individuals, allowing for a total of 43,954 observations. Appendix Figures A1 shows the number of respondents by the number of repeated measures. For example, 195 respondents have 11 observations. While panel attrition is evident, the sample is representative of the political distribution in the Netherlands as measured by voting behaviour.

Data were collected via the Internet, allowing participants to share their political attitudes without pressure to respond with socially desirable answers. Respondents' tendencies to adjust their actual perception to what they perceive as a socially desirable answer, compared with what they really think, is a major problem in face‐to‐face surveys; particularly when measuring support for stigmatised radical parties (Tourangeau et al., 2000). This advantage is confirmed by Rekker et al. (2020) showing that the LISS data is representative of the political distribution of the Netherlands, particularly regarding the less socially desirable RWP support, compared with a face‐to‐face survey. Appendix Table A1 demonstrates the representativeness of each subsample used in this study, in each of the five election cycles covered by the panel. It is evident that the subsample retains its representativeness of the actual Dutch election results. Major discrepancies, not surprisingly, appear mostly among small parties. The PVV is quite well‐represented, with the sampling ranging from 71% (for the 2017 elections) to 84% (for the 2010 and 2012 elections) of their actual voting share.

To test our hypotheses, we employ two‐level hierarchical linear fixed‐effects regression models, fitting time‐points nested within individuals, allowing fixed‐effects for years. The use of fixed‐effects models grants significant advantages when utilising our longitudinal data. Fixed‐effects removes the personal across‐time mean of each person in the sample from each time‐point observation. Thus, the model controls for all possible time‐invariant mediating variables, such as birth cohort, place of birth, sex, and early‐life socialisation. The coefficients are to be interpreted as the effect of change of the independent variable on the dependent variable—sympathy for right‐wing populism, although we cannot exclude the role of not included time varying variables. Moreover, our models also incorporate fixed effects for years, and allow them to change between men and women. This allows the models to control for most contextual social and political changes occurring in the Netherlands in the span of the data, including, among others, any increase in the number of immigrants, any change in the composition or rhetoric of RWP parties, and any macro‐economic change. The models take into account the possible different effects these contextual events may have on men and women separately. Thus, these fixed‐effects models imply that many alternative explanations of RWP support are already accounted for.

To test hypotheses H1a/b, we utilise Equation (1), presented here as a formal expression:

(1) $${\ddot{y}}_{it}=\bar{\beta_{0}}{\bar{YEAR}}_{t}+\sum_{k}\beta_{k}{\ddot{X}}_{kit}+{\ddot{\epsilon }}_{it}$$

For every individual *i* in time *t*, ${\ddot{y}}_{it}$ is the predicted change in the dependent variable, attitude towards RWP parties, between the mean value of individual *i*, to the value at time *t*; $\bar{\beta_{0}}$ is the vector of fixed‐effects for the years and ${\bar{YEAR}}_{t}$ is the vector of year at time *t*; *β*_*k*_ is the coefficient of the *k*'th independent variable; ${\ddot{X}}_{kit}$ is the change in value of the *k*'th independent variable between the mean for individual *i* and the value at time *t*; and, finally, ${\ddot{\epsilon }}_{it}$ is the change in error between the mean error of individual *i* and the error in time *t*, under the assumption that ${\ddot{\epsilon }}_{it}∼N\left(0,\sigma_{it}\right)$.

To test hypotheses 2a/b1/b2/c, we add an interaction between gender and income, as well as between gender, on the one hand, and labour market variables, age, number of children, and the year fixed effects, on the other. The interactions used are those that remain after dropping the insignificant interactions one by one in the full interaction model. This allows us to test for the moderating and mediating effects of gender. To test hypothesis 2c we utilise the Z‐test of cross‐model differences, as suggested by Mize et al. (2019), under assumptions of non‐negatively correlated standard errors for the marginal effects of women's personal and household income. Hypotheses three is tested for a subsample of non‐single‐sex couples, by adding a 1^st^ order and 2^nd^ order interaction terms with a new dummy variable. This new dummy variable defines for each household whether, on the average of all time points, the woman is the highest earner in the household (=1) or the man is (=0). The interactions are with the personal income variables, and with the interactions between personal income and gender.

## 6 FINDINGS

### 6.1 Income change and sympathies towards RWP parties

**FIGURE 2:**
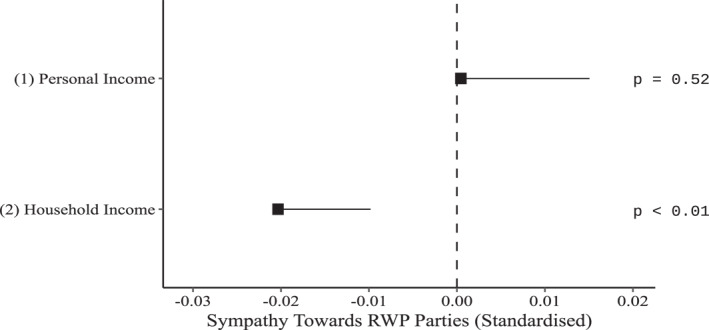
Forest‐plot showing the marginal effects (with one‐tailed confidence intervals [95%]), and one‐tailed *p*‐values of personal and household income (see Appendix Table A2 for the regression coefficients).

We hypothesise that income change, both at (a) the personal and at (b) the household level, is negatively related to change in sympathy towards RWP parties. Appendix Table A2 presents the coefficients of two multi‐level fixed‐effects models, examining the effect of income, transformed by the natural logarithm, and standardised on our subsample, on sympathies towards RWP parties. The models control for the year, demographic variables (age, number of children, co‐habiting partner, density of area of domicile), having experienced unemployment, level of education, and all time‐invariant individual properties. All coefficients of the regression are presented in Model 1a and 1b of Appendix Table A8. Figure 2 presents the marginal effects of personal income and household income, based on models 1 and two of Appendix Table A2.

We note from Model 1 that personal income is not linked with sympathy towards RWP parties. Thus, we fail to confirm hypothesis H1a.

Models 2 tests for hypothesis H1b: household income change is negatively related with sympathies towards RWP parties. Here, consistent with the hypothesis, we find that income on the household level is negatively related with sympathies towards RWP parties (Table A2, Model 2: *b* = −0.020, *p* < .01). This means that every standard deviation [SD] for log income, there is a fall of 0.020 SDs in sympathies towards RWP parties, net of all controls. Thus, our findings support hypothesis H1b.

We note that effect sizes are small, which is to be expected. The effect sizes here are consistent with the small effect sizes in previous works documenting the relationship between economic variables and political attitudes (e.g., Abou‐Chadi & Kurer, 2021; Burgoon et al., 2019; Gidron & Mijs, 2019; Hartmann et al., 2022). We suggest three reasons for this. Firstly, as suggested by Halikiopoulou and Vlandas (2020), RWP parties have both core and peripheral supporters. While the small number of core supporters are motivated by cultural grievances and nativism, a much larger number of peripheral supporters are motivated by economic concerns. While core supporters are more likely to support right‐wing populism, the much larger group of peripheral supporters, who are motivated by economic grievances, are less likely to support right‐wing populism, but aggregate into the largest support base. Thus, small effect sizes may predict great shifts in aggregate. Secondly, the models predict the causes of change by tracing them within a brief time span. However, the resulting attitude change may be the tail end of a process that is long term (see Hartmann et al., 2022). Thirdly, income change itself is not immediately connected to attitudinal change. The connection may be moderated by perceptions, personal circumstances, and more. However, when controlling for social trust, satisfaction with the government, attitudes towards immigrants, and left/right political alignment, the results remain robust (Appendix Table A5, Model K; this is true for all models in this paper; see Appendix Tables A5, A6 and A7 and their descriptions).

Furthermore, we note that when analysing income change separately for decreasing income (see Table A5, Model H), increasing income (see Table A5, Model I), and voting for RWP parties instead of sympathies towards them (see Table A5, Model J), the results point to similar patterns, albeit the effects may be insignificant due to a much‐reduced number of observations.^4^

### 6.2 The moderating effect of gender

After establishing that income change, on the household level, is related to change in sympathy towards right‐wing populism, we turn to gender to understand why personal income appears not to be related to RWP sympathy. The theory of gendered socialisation, whereby men are more sensitive than women to their personal economic situation when determining their political attitudes and behaviour, may be the reason we see this difference. Women are expected to accentuate social context in their political motivations.

We now examine our second set of hypotheses, which explore how gender moderates the link between income change and shifts in sympathy towards RWP parties. Regarding personal income, we expect this link to be stronger for men, and weaker for women (H2a). Concerning household income, as discussed above, the socialisation theory suggests two contrasting hypotheses (H2b1/2): on the one hand, women may accentuate household income *less* than men due to their (women's) focus on sociocultural context (H2b1). On the other hand, women may prioritise household income *more* than men due to their (women's) greater emphasis on social context (H2b2). Finally, for women, we expect that changes in personal income have less impact on their RWP sympathies than changes in household income (H2c).

**FIGURE 3:**
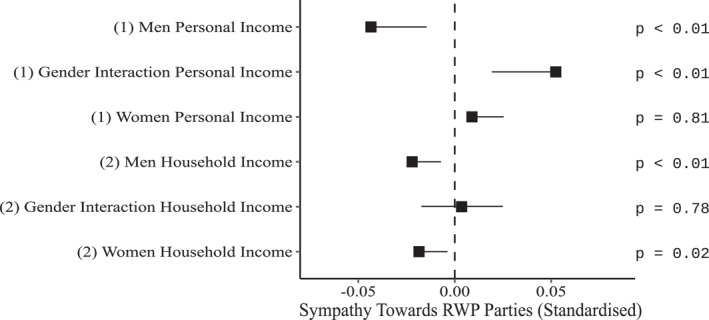
Forest‐plot showing the marginal effects (with one‐tailed confidence intervals [95%]), and one‐tailed *p*‐values of personal and household income by gender and the interactions between income and gender (see Appendix Table A3 for the regression coefficients).

Appendix Table A3 shows the coefficients of our fixed‐effects multi‐level models predicting sympathy towards RWP parties by personal and household income. All models in Appendix Table A3 include controls for years, and all other controls as the models presented in Table A2 (all omitted coefficients appear in Appendix Table A8, Models 2a and 2b). Education, unemployment, and year are allowed to change by gender, and all other demographic controls that had a significant interaction with gender are included as well. Figure 3 presents the marginal effects of personal and household income by gender and the interactions between income and gender for the models presented in Appendix Table A3.

Model 1 examines the link between personal income and sympathies towards RWP parties. Men's personal income coefficient is negative and significant (Table A3, Model 1: *b* = −0.043, *p* < .01). As hypothesised, there is an interaction between gender and income (Table A3, Model 1: *b* = 0.052, *p* < .01), with women's coefficient being small and insignificant (Model 1: *b* = −0.043 + 0.052 = 0.009, *p* > .10).^5^ This means that for every increase of one SD, men's sympathies towards right‐wing populism decrease by 0.043 SDs, net of all controls. Thus, our findings support hypothesis H2a.

After establishing that the effect of personal income is gender‐dependent, we turn to household income. In Model 2, we see that for both men (*b* = −0.022, *p* < .01) and women (*b* = −0.022 + 0.004 = −0.018, *p* < .05), household income is negatively related to sympathies for RWP parties. This means that for every increase of one SD in the household income, men's sympathies towards RWP parties decrease by 0.022 SD, and women's sympathies decrease by 0.018 SD, net of all controls. The interaction term indicates no statistically significant difference between men's and women's coefficients (*b* = 0.004, *p* > .10). Thus, contrary to hypotheses H2b1/2, we find no gender moderation of the link between household income and RWP sympathies. The effect of household income change on RWP sympathies appears no different between men and women. It may be that the two contradictory effects are superimposed, thus obscuring any gender moderation.

Testing for hypothesis H2c, we find a significant difference for women between the effect of personal income and the effect of household income (*Z* ≥ 2.01; *p* < .05),^5^ corroborating the hypothesis. Household income matters more to women than personal income when it comes to sympathy towards RWP parties. This indicates that women's sensitivity to context, as hypothesised in the socialisation theory, includes the economic situation of their own household.

We apply several robustness checks on this model (see Appendix Table A6 and its description). We see the same patterns replicating for increasing (Table A6, Model I) and decreasing incomes (Table A6, Model H) separately, and for voting instead of sympathy towards right‐wing populism (Table A6, Model J). However, as before, due to loss of many observations, some coefficients may be marginally significant or insignificant.^7^

In the next section, we examine the economic dynamics within households to test an alternative explanation for the gender difference in the effect of personal income.

### 6.3 Household economic dynamics

**FIGURE 4:**
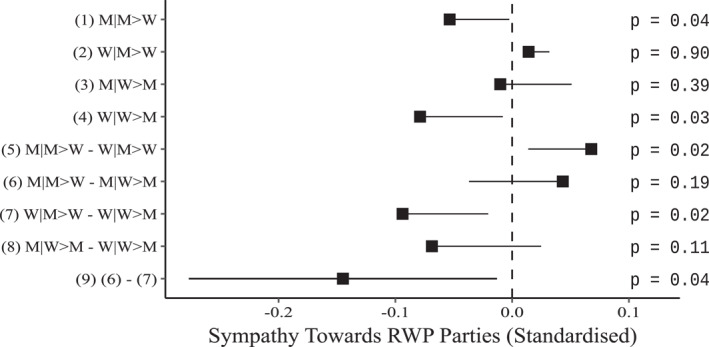
Forest‐plot showing the marginal effects (with one‐tailed confidence intervals [95%]), and one‐tailed *p*‐values of personal income change on right‐wing populist sympathy (see Appendix Table A4 for the regression coefficients of this model). Coefficients interpretations: (1)M|M > W: *Income change* (income coefficient for men, when men earn more).(2)W|M > W: *Income change* + *Income change* × *Women* (income coefficient for women when men earn more).(3)M|W > M: *Income change* + *Income change* × *Women earn more* (income coefficient for men when women earn more).(4)W|W > M: *Income change* + *Income change* × *Women* + *Income change* × *Women earn more* + *Income change* × *Women* × *Women earn more* (income coefficient for women when women earn more).(5)M|M > W – W|M > W: *Income change* × *Women* (the difference between coefficients (1) and (2)).(6)M|M > W – M|W > M: *Income change* × *Women earn more* (the difference between coefficients (1) and (3)).(7)W|M > W – W|W > M: *Income change* × *Women earn more* + *income change* × *Women* × *Women earn more* (the difference between coefficients (2) and (4)).(8)M|W > M – W|W > M: *Income change* × *Women* + *Income change* × *Women* ×  *Women earn more* (the difference between coefficients (3) and (4)).(9)(6) and (7): *Income change* × *Women* × *Women earn more* (the difference between coefficients (6) and (7)); The difference between (1) and (4) is statistically insignificant (Wald‐test F(1, 5810) = 0.22, *p* = .64).

Having established that men's, not women's, personal income change is related to change in RWP sympathy, we test an alternative theory that may explain this gendered pattern: the economic dominance theory. If women's RWP sympathy is influenced by their personal income solely when they are the primary earners in the household, this implies that women's economic position within the household, rather than socialisation, accounts for the average lack of impact of women's personal income. Since most Dutch women earn less than their husbands, this may explain the gendered patterns observed in the previous section (see Figure 3). Thus, we test hypothesis H3 which posits that the change in personal income of the partner whose income contributes most to household income is negatively related to RWP sympathy. Appendix Table A4 displays the coefficients of a fixed‐effects multi‐level model predicting RWP sympathy by personal income, interacted with gender and whether the woman is the highest earner in the household (a dummy variable). All the models include all controls as in the models presented in Figure 3 (omitted coefficients appear in Appendix Table A8, Model 3a). Figure 4 plots the marginal effects of personal income on RWP sympathy for the models presented in Appendix Table A4.

Men's estimate for personal income is negative and significant (b(1) = −0.053, *p* < .05). This means that for every increase of one SD for men outearning their partner, there is a decrease of 0.053 SD in RWP sympathies, net of all controls. We find an interaction between gender and income (b(5) = 0.068, *p* < .01), which leads to women's income being insignificant (b(2) = −0.053 + 0.068 = 0.014, *p* > .10) when the man is the highest earner in the household.^7^ Thus, we see that in the case of men outearning their partner, there is no personal income effect on RWP sympathy for women. These are the same results found in the previous analysis, presented in Figure 3, which is plausible, given that, in the Netherlands, most women earn less than their partners.

However, in households where women outearn their partners, we observe a significant effect of women's personal income on RWP sympathy (b(4) = −0.053 + 0.068 + 0.043 + −0.136 = −0.079, *p* < .05). This indicates that for women earning more than men, an increase of one SD in income corresponds to a decrease of 0.079 SD in RWP sympathy, net of all controls. The disparity between the income effect for women when they are the highest earner in the household, versus when their partner is, is significant (b (7) = 0.043–0.136 = −0.093, *p* < .05). This suggests that the effect of personal income on RWP sympathy exists for women only when they outearn their male partner. However, no such distinction was observed for men. While there are indications that the link between personal income and RWP sympathy does not exist for men when they earn less than their partners (b (3) = −0.053 + 0.043 = −0.010, *p* > .10), the difference coefficient between the effect of men's income when they earn more than their partner, versus the effect of income when men earns less is insignificant (b(6) = 0.043, *p* > .10). Correspondingly, the difference between the income effect of women versus men − when women earn more than their partner − is also insignificant (b(8) = −0.069, *p* > .10). Importantly, the difference between the coefficient for men, when the man is the higher earner, and the coefficient for women, when the woman is the higher earner is insignificant (*b* = −0.053 to −0.079 = 0.027, Wald test F(1, 5810) = 0.22, *p* > .10). Finally, the disparity between the effect of the difference for men, whether they are the highest earner in the household or the woman is (coefficient no. 6), and the effect of the difference for women, whether they are the highest earner or the man is (coefficient no. 7), is significant (b(9) = −0.136, *p* < .05). In other words, women are more affected by being the highest earner than men are.

Our findings partially support hypothesis 3 regarding women, but not regarding men. When women earn less than their partner, change in their personal income does not affect their RWP sympathy, but when they outearn their partner, it does. For men, however, we find no difference in the relationship between income change and RWP sympathy, whether they earn less or more than their partner.

The results partially confirm the economic dominance theory, at least for women. More importantly, they challenge the ‘economic man’ and ‘social woman’ theory proposed by Gidengil (1995), demonstrating that women do behave ‘economically’. Conversely, for men, their partner's earnings do not appear to influence the relationship between their personal income and RWP sympathy. Indeed, we find that the effect of being the highest earner affects women much more than it does men. This disparity lends credence to the socialisation theory, indicating that women derive political attitudes not only from their personal economic status, but also from their social context, whereas men base their attitudes on their own economic situation alone—their partner's earning do not seem to sway them.

We conduct several robustness tests, detailed in Appendix Table A7 and its description. We note that separate analyses for increasing (Model F) and decreasing (Model E) income yield similar patterns as our main analysis, albeit insignificant (possibly due to loss of observations).^8^

## 7 DISCUSSION AND CONCLUSIONS

In this paper we set out to disentangle the complex relationship between income and sympathy towards RWP parties. Utilising fixed‐effects multilevel models on the Dutch LISS panel survey, with data ranging from 2007 to 2021, we demonstrate, for the first time, a negative correlation between objective income change over time and shifts in RWP sympathy. Our results show that for both men and women, household income change is negatively correlated with sympathies for right‐wing populism. However, we observe that changes in personal income appear to only affect men's RWP sympathies. We examine whether this gender discrepancy arises from gendered socialisation, whereby women, compared with men, are motivated more by their sociocultural context than by their own economic status, leading to divergent behaviour even when sharing the same economic attributes with men (e.g., Chaney et al., 1998; Coffé, 2013; Donovan, 2023; Gidengil, 1995; Gilligan, 1982; Sipma et al., 2023a; Welch & Hibbing, 1992); or whether it can be attributed to economic dominance, which centres on intra‐household economic dynamics, suggesting household members align political positions based on the economic status of the highest earner (De Graaf & Heath, 2023; Strøm, 2014). The prevalence of the male breadwinner model in the Netherlands could offer one way of explaining the gender disparity in reactions to personal income change.

Indeed, we find that when women are the primary earners in the household, they experience the anticipated negative correlation between personal income and RWP sympathies, a finding which corroborates the economic dominance hypothesis. Yet, for men, we find no significant difference in how their RWP sympathies are affected by personal income, whether they are the highest earners in the household or not, which in turn lends support to the socialisation theory. We do not discount the possibility that a self‐selection process may be at play here, whereby women who emphasise their economic standing also tend to outearn their partners. Further longitudinal studies on the impact of individual‐level economic change on RWP support are warranted to investigate this.

Our results then clearly demonstrate the correlation between objective individual‐level income fluctuations and shifts in support for right‐wing populism. Our focus on income was motivated by it being one of the most important indicators of economic standing, alongside occupation and employment status. However, as we discussed in our review, previous research attempting to link objective income changes with RWP support has only yielded conflicting results (Gidron & Mijs, 2019; Hartmann et al., 2022). Notably, only the study by Gidron and Mijs (2019) utilised panel data tracking changes in political attitudes alongside income changes. Prior studies on the impact of income on RWP support often concentrated on relative and subjective measures of income instead (Burgoon et al., 2019; Kurer, 2020; Sipma et al., 2023b). While this line of research is promising, it remains important to understand the relationship between, on the one hand, objective macro‐level economic shifts and RWP support as established in prior research (e.g., Anelli et al., 2021; Autor et al., 2013; Colantone & Stanig, 2019; Guriev & Papaioannou, 2022; Scheiring et al., 2024), and, on the other hand, objective *micro*‐level economic changes, with objective income change being of particular significance. Only through elucidating micro‐level mechanisms can we substantiate the potential existence of the macro‐level connection between economic change and the rise of right‐wing populism (see Coleman, 1990).

While illustrating the connection between objective income change and RWP support, this paper improves upon previous research both methodologically and theoretically. Crucially, we demonstrate that to understand the relationship between individual‐level economic factors and RWP support, one must not only examine the relationship through time, but also consider gender and, in particular, the imbalance in earnings between men and women within households. Employing longitudinal data enabled us to trace the trajectories of individual attitudes through time and scrutinise the long‐standing claim that links income change to RWP support more rigorously than previously possible. When considering gender and within‐household inequality, our data indeed suggest that individuals change their minds regarding right‐wing populism when they face income change. This relationship is negative. Those experiencing an increase in income, tend to display diminishing sympathy towards right‐wing populism, while those who suffer a decrease in income, tend to become more sympathetic towards it. This nuanced understanding could not have been gleaned from cross‐sectional data alone. Furthermore, these results underscore how enhancing material conditions can diminish the allure of RWP parties.

## CONFLICT OF INTEREST STATEMENT

None.

## Supporting information

Supporting Information S1

## Figures and Tables

**FIGURE A2:**
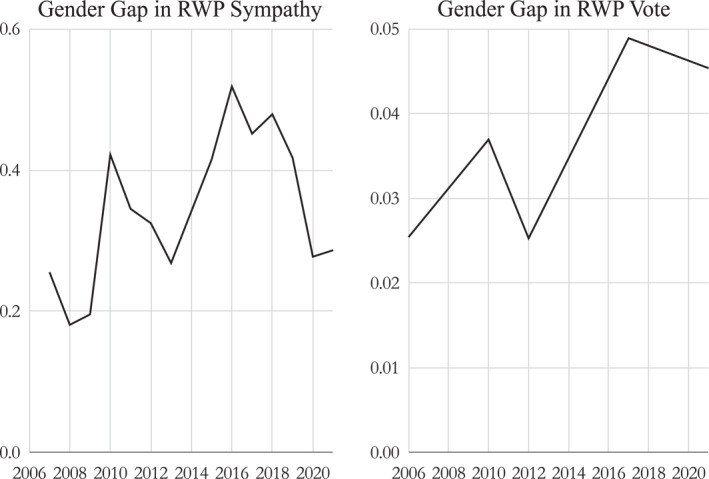
Gender gap (men minus women) through time of sympathies towards right‐wing populist parties and vote for RWP parties.

## Data Availability

The data underlying this article were provided by CentERdata (Tilburg University, the Netherlands) under licence. The data are available at https://www.dataarchive.lissdata.nl/.

## ENDNOTES

1 According to LISS data, during the 2023 election 53% of the PVV voters were male and 47% female. Of the elected people in Parliament, among the 37 PVV members 7 are female. Hence, the Party is a male dominated political party and this has hardly changed over time. See Appendix Figure A2, which shows the change over time in the gender gap in RWP sympathy and RWP vote. The gap is sustained through time.

3 Alpha Cronbach was calculated for each year separately on the combined variable, as parties and their leaders changed during the years included in the data. The alpha coefficient ranges between .86 in 2021 and .96 in 2011.

4 Furthermore, we see the same patterns replicated when testing only sympathy towards the PVV (Table A5, Model B); testing only sympathy towards the FvD (Table A5, Model C); testing only those who are gainfully employed (Table A5, Model D); testing only those who are in a partnership (Table A5, Model E); testing only those who are not in partnership (Table A5, Model F); testing only those who are single (Table A5, Model G); testing for gross instead of net log income (Table A5, Model L); and finally testing only those who are both in gainful employment and in partnership (Table A5, Model M).

5 The significance level and the standard error were obtained via a reverse analysis, flipping the gender coefficient between male and female. The significance test of this main effect is the same as a Wald test.

7 Furthermore, we see the same patterns replicated when testing only sympathy towards the PVV (Table A6, Model B); testing only sympathy towards the FvD (Table A6, Model C); testing only those who are gainfully employed (Table A6, Model D); testing only those who are in a partnership (Table A6, Model E); testing only those who are not in partnership (Table A6, Model F); testing only those who are single (Table A6, Model G); testing with controls for trust, satisfaction of government, left‐right political alignment and attitudes towards immigrants (Table A6, Model K); testing for gross instead of net log income (Table A6, Model L); and finally testing only those who are both in gainful employment and in partnership (Table A6, Model M).

8 The significance level and the standard error were obtained via a reverse analysis, flipping the gender coefficient between male and female. The significance test of the new main effects is the same as a Wald test.

## APPENDIX A

**TABLE A1:** Our sample for each election cycle, compared with actual election results.

Election cycle	2006				2010				2012				2017				2021			
Party	Real	Sample	Ratio	Gap	Real	Sample	Ratio	Gap	Real	Sample	Ratio	Gap	Real	Sample	Ratio	Gap	Real	Sample	Ratio	Gap
PVV	5.89%	4.66%	0.79	−1.23%	15.45%	12.94%	0.84	−2.51%	10.08%	8.47%	0.84	−1.61%	13.06%	9.29%	0.71	−3.77%	10.79%	8.17%	0.76	−2.62%
FvD	‐	‐	‐	‐	‐	‐	‐	‐	‐	‐	‐	‐	1.78%	2.18%	1.22	0.40%	5.02%	2.65%	0.53	−2.37%
JA21	‐	‐	‐	‐	‐	‐	‐	‐	‐	‐	‐	‐	‐	‐	‐	‐	2.37%	3.27%	1.38	0.90%
LPF	0.21%	0.60%	2.86	0.39%	‐	‐	‐	‐	‐	‐	‐	‐	‐	‐	‐	‐	‐	‐	‐	‐
1NL	0.64%	0.31%	0.48	−0.33%	‐	‐	‐	‐	‐	‐	‐	‐	‐	‐	‐	‐	‐	‐	‐	‐
**Total RWP**	**6.74%**	**5.57%**	**0.83**	**−1.17%**	**15.45%**	**12.94%**	**0.84**	**−2.51%**	**10.08%**	**8.47%**	**0.84**	**−1.61%**	**14.84%**	**11.47%**	**0.77**	**−3.37%**	**18.18%**	**14.09%**	**0.78**	**−4.09%**
VVD	14.67%	15.17%	1.03	0.50%	20.49%	22.38%	1.09	1.89%	26.58%	25.73%	0.97	−0.85%	21.29%	20.80%	0.98	−0.49%	21.87%	21.45%	0.98	−0.42%
PvdA	21.19%	20.00%	0.94	−1.19%	19.63%	17.79%	0.91	−1.84%	24.84%	22.92%	0.92	−1.92%	5.70%	7.95%	1.39	2.25%	5.73%	7.72%	1.35	1.99%
CDA	26.51%	21.74%	0.82	−4.77%	13.61%	10.24%	0.75	−3.37%	8.51%	6.73%	0.79	−1.78%	12.38%	10.18%	0.82	−2.20%	9.50%	9.59%	1.01	0.09%
SP	16.58%	17.99%	1.09	1.41%	9.82%	11.30%	1.15	1.48%	9.65%	12.08%	1.25	2.43%	9.09%	9.60%	1.06	0.51%	5.98%	7.01%	1.17	1.03%
D66	1.96%	3.77%	1.92	1.81%	6.95%	8.89%	1.28	1.94%	8.03%	9.73%	1.21	1.70%	12.23%	13.56%	1.11	1.33%	15.02%	16.15%	1.08	1.13%
GL	4.60%	6.76%	1.47	2.16%	6.67%	8.94%	1.34	2.27%	2.33%	3.29%	1.41	0.96%	9.13%	11.25%	1.23	2.12%	5.16%	5.85%	1.13	0.69%
CU	3.97%	4.88%	1.23	0.91%	3.24%	3.74%	1.15	0.50%	3.13%	3.38%	1.08	0.25%	3.39%	4.34%	1.28	0.95%	3.37%	4.12%	1.22	0.75%
SGP	1.56%	1.56%	1.00	0.00%	1.74%	1.51%	0.87	−0.23%	2.09%	1.50%	0.72	−0.59%	2.08%	1.55%	0.75	−0.53%	2.07%	1.51%	0.73	−0.56%
PvdD	1.83%	1.85%	1.01	0.02%	1.30%	1.38%	1.06	0.08%	1.93%	2.02%	1.05	0.09%	3.19%	3.71%	1.16	0.52%	3.84%	4.63%	1.21	0.79%
50+	‐	‐	‐	‐	‐	‐	‐	‐	1.88%	2.24%	1.19	0.36%	3.11%	3.18%	1.02	0.07%	1.02%	1.35%	1.32	0.33%
DENK	‐	‐	‐	‐	‐	‐	‐	‐	‐	‐	‐	‐	2.06%	0.65%	0.32	−1.41%	2.03%	1.00%	0.49	−1.03%
Volt	‐	‐	‐	‐	‐	‐	‐	‐	‐	‐	‐	‐	‐	‐	‐	‐	2.42%	2.93%	1.21	0.51%
BBB	‐	‐	‐	‐	‐	‐	‐	‐	‐	‐	‐	‐	‐	‐	‐	‐	1.00%	0.68%	0.68	−0.32%
BIJ1	‐	‐	‐	‐	‐	‐	‐	‐	‐	‐	‐	‐	‐	‐	‐	‐	0.84%	0.42%	0.50	−0.42%
N (unique voters)	‐	4159	‐	‐	‐	3823	‐	‐	‐	4411	‐	‐	‐	4313	‐	‐	‐	3109	‐	‐

*Note*: Real = the percent of total votes won in the elections; Sample = the percent of those reported to have voted for this party in the sample; Ratio = the ratio between the sample vote and real vote: ratio > 1: oversampling; ratio < 1: undersampling; Gap = the gap between the sample vote and real vote.

**TABLE A2:** Standardised coefficients of a fixed‐effects hierarchical linear regression predicting sympathies towards RWP parties.

Model	(1)	(2)
Income		
Personal	0.000	
	(0.009)	
Household		−0.020**
		(0.006)
Adjusted r‐squared	0.0373	0.0376
AIC	55053.95	55038.93
No. of individuals	7780	
No. of data points	43,954	

*Note*: Standard errors are clustered for individuals; all models control for level of education, unemployment, socio‐demographic variables and year fixed‐effects. Educational level divides into: primary, vocational, general secondary and academic education; unemployment: has been unemployed in the last year; demographics contain: number of children, age, population density of area of domicile and cohabiting partner. Alternative models excluding education and unemployment lead to the same results.

***p* < .01, **p* < .05, +*p* < .10, one‐tailed tests.

**TABLE A3:** Standardised coefficients of a fixed‐effects hierarchical linear regression predicting attitudes towards RWP parties, with gender moderating effects.

Model	(1)	(2)
Income		
Personal	−0.043**	
	(0.018)	
Personal × Woman	0.052**	
	(0.020)	
(Women's personal coefficient)	0.009	
	(0.010)	
Household		−0.022**
		(0.009)
Household × Woman		0.004
		(0.013)
(Women's household coefficient)		−0.018*
		(0.009)
Adjusted r‐squared	0.03872	0.03880
AIC	55010.12	55006.2
No. of individuals	7780	
No. of data points	43,954	

*Note*: Standard errors are clustered for individuals; all models control for level of education (allowed to change by gender), unemployment (allowed to change by gender), socio‐demographic variables (significant coefficients allowed to change by gender) and year (allowed to change by gender). Educational level divides into: primary, vocational, general secondary and academic education; unemployment: has been unemployed in the last year; demographics contain: number of children, age, population density of area of domicile and cohabiting partner. Alternative models excluding education and unemployment lead to the same results.

***p <* .01, **p <* .05, +*p <* .10, one‐tailed tests; Household × Woman is tested using a two‐tailed test.

**TABLE A4:** Standardised coefficients of a fixed‐effects hierarchical linear regression predicting attitudes towards RWP parties, allowing income to change by gender and by who earns more in the household.

	Figure 4 Legend	Personal
Income		
(1) Personal	M|M > W	−0.053*
		(0.031)
(2) Personal + Personal × Woman	W|M > W	0.014
		(0.011)
(3) Personal + Personal × Woman earn more	M|W > M	−0.010
		(0.037)
(4) Personal + Personal × Woman + Personal × Woman earn more + Personal × Woman × Women earn more	W|W > M	−0.079*
		(0.043)
(5) Personal × Woman	M|M > W – W|M > W	0.068*
		(0.033)
(6) Personal × Woman earn more	M|M > W – M|W > M	0.043
		(0.049)
(7) Personal × Woman earn more + Personal × Woman × Woman earn more	W|M > W – W|W > M	−0.093*
		(0.044)
(8) Personal × Woman + Personal × Woman × Woman earn more	M|W > M – W|W > M	−0.069
		(0.057)
(9) Personal × Woman × Woman earn more	(6) – (7)	−0.136*
		(0.066)
Adjusted r‐squared		0.04367
AIC		42661.0
No. of individuals		5811
No. of data points		33,965

*Note*: Standard errors are clustered for individuals; all models control for level of education (allowed to change by gender), unemployment (allowed to change by gender), socio‐demographic variables (significant coefficients allowed to change by gender) and year fixed‐effects (allowed to change by gender). Educational level divides into: primary, vocational, general secondary and academic education; unemployment: has been unemployed in the last year; demographics contain: number of children, age, population density of area of domicile and cohabiting partner. Alternative models excluding education and unemployment lead to the same results. Coefficient interpretation: (1) Men, when men earn more; (2) Women, when men earn more; (3) Men, when women earn more; (4) Women, when women earn more; (5) Difference between (1) and (2); (6) Difference between (1) and (3); (7) Difference between (2) and (4); (8) Difference between (3) and (4); (9) Difference between (6) and (7).

***p* < .01, **p* < .05, +*p* < .10, one‐tailed tests; Coefficient 9 is tested using a two‐tailed test.

**TABLE A5:** Standardised coefficients of a fixed‐effects hierarchical linear regression predicting attitudes towards RWP parties.

Robustness test	Dependent variable: Sympathies towards RWP parties		Dependent variable: PVV sympathy		Dependent variable: FvD sympathy		Subsample: Gainfully employed only		Subsample: In partnership only		Subsample: Non‐partner only		Subsample: Household of singles only		Subsample: Decreasing income		Subsample: Increasing income		Dependent variable: RWP voting		Control of social trust, satisfaction of government, left‐right political alignment, and attitudes towards immigrants		Independent variable: Gross log income change		Subsample: Gainfully employed and in partnership only	
Model	(A)		(B)		(C)		(D)		(E)		(F)		(G)		(H)		(I)		(J)		(K)		(L)		(M)	
	(1)	(2)	(1)	(2)	(1)	(2)	(1)	(2)	(1)	(2)	(1)	(2)	(1)	(2)	(1)	(2)	(1)	(2)	(1)	(2)	(1)	(2)	(1)	(2)	(1)	(2)
Income																										
Personal	0.000		−0.003		−0.035*		0.000		0.003		−0.015		−0.034+		0.004		0.005		−0.008+		0.001		0.001		0.003	
	(0.009)		(0.008)		(0.020)		(0.009)		(0.010)		(0.018)		(0.023)		(0.011)		(0.014)		(0.006)		(0.009)		(0.009)		(0.010)	
Household		−0.020**		−0.021**		−0.029*		−0.019*		−0.033**		−0.012+		‐		−0.025**		−0.009		−0.005		−0.021**		−0.019**		−0.033**
		(0.006)		(0.006)		(0.014)		(0.007)		(0.011)		(0.007)		‐		(0.008)		(0.010)		(0.004)		(0.007)		(0.006)		(0.013)
Adjusted r‐squared	0.0373	0.0376	0.0285	0.0289	0.2542	0.254	0.0375	0.0378	0.0432	0.0437	0.0261	0.0262	0.0269	‐	0.0376	0.0379	0.0375	0.0374	0.0131	0.0134	0.0690	0.0694	0.0373	0.0376	0.0436	0.0440
AIC	55053.95	55038.93	50966.98	50948.96	17370.45	17369.62	53273.27	53260.75	39855.01	39840.38	15165.08	15163.22	9617.649	‐	34669.11	33290.76	22168.41	21889.12	−9962.494	−9915.327	44507.26	44492.58	55053.97	55042.04	38225.39	38212.79
Variance components																										
*Sd(u)*	0.891	0.890	0.902	0.901	3.032	3.031	0.889	0.889	0.896	0.894	0.907	0.908	0.921	‐	0.897	0.887	0.883	0.893	0.312	0.313	0.812	0.811	0.891	0.890	0.895	0.894
*Sd(e)*	0.499	0.499	0.476	0.476	0.573	0.573	0.498	0.498	0.498	0.498	0.500	0.500	0.487	‐	0.502	0.494	0.496	0.507	0.196	0.196	0.479	0.479	0.499	0.499	0.497	0.497
No. of individuals	7780		7780		3889		7520		5475		2305		1525		4861	4819	3182	2973	4173	4117	7487		7780		5244	
No. of data points	43,954		43,954		12,756		42,645		31,755		12,199		8072		27,396	27,033	17,874	16,993	11,431	11,248	38,693		43,954		30,555	

*Note*: Standard errors are clustered for individuals; all models control for level of education, unemployment, socio‐demographic variables and year fixed‐effects. Educational level divides into: primary, vocational, general secondary and academic education; unemployment: has been unemployed in the last year; demographics contain: number of children, age, population density of area of domicile and cohabiting partner. Alternative models excluding education and unemployment lead to the same results.

Models descriptions: Model A: The main model of this paper, as presented in Table A2 and Figure 2. Model B: The dependent variable is sympathy towards PVV and Wilders only. Model C: The dependent variable is sympathy towards FvD and Baudet only. Model D: Only respondents who have an income on average in all time points. Model E: Only respondents who are in a partnership (either married or co‐habiting) in most of the time points. Model F: Only respondents who are not in partnership (either married or co‐habiting) in most of the time points. Model G: Only respondents who are the sole member of their household in most of the time points. Model H: Only respondents whose income trajectory through time is negative. Model I: Only respondents whose income trajectory through time is positive. Model J: Change in dependent variable to voting for RWP parties. A fixed‐effect multilevel linear probability model predicting voting behaviour (see Sipma et al., 2023a for a similar model and a discussion of its advantages against Logit model). Voting behaviour was taken from the closest time point to an election, and income is average from preceding time since last election. Thus we have only four times points, as the number of elections. Model K: Same as Model A, but with extra controls for social trust, satisfaction of government, left‐right political alignment, and attitudes towards immigrants. Model L: Personal and household income are gross income instead of net income. Model M: Only respondents who have an income on average through all time points and who are also in partnership (either married or co‐habiting) in most of the time points.

**p* < .01, **p* < .05, +*p* < .10, one‐tailed tests.

**TABLE A6:** Standardised coefficients of a fixed‐effects hierarchical linear regression predicting attitudes towards RWP parties, with gender moderating effects.

Robustness test	Dependent variable: Sympathies towards RWP parties		Dependent variable: PVV sympathy		Dependent variable: FvD sympathy		Subsample: Gainfully employed only		Subsample: In partnership only		Subsample: Non‐partner only		Subsample: Household of singles only		Subsample: Decreasing income		Subsample: Increasing income		Dependent variable: RWP voting		Control of social trust, satisfaction of government, left‐right political alignment, and attitudes towards immigrants		Independent variable: Gross log income change		Subsample: Gainfully employed and in partnership only	
Model	(A)		(B)		(C)		(D)		(E)		(F)		(G)		(H)		(I)		(J)		(K)		(L)		(M)	
	(1)	(2)	(1)	(2)	(1)	(2)	(1)	(2)	(1)	(2)	(1)	(2)	(1)	(2)	(1)	(2)	(1)	(2)	(1)	(2)	(1)	(2)	(1)	(2)	(1)	(2)
Income																										
Personal	−0.043**		−0.038**		−0.087**		−0.043**		−0.047*		−0.044*		−0.052*		−0.040+		−0.039*		−0.024+		−0.050**		−0.043**		−0.047*	
	(0.018)		(0.018)		(0.041)		(0.018)		(0.027)		(0.023)		(0.025)		(0.027)		(0.023)		(0.018)		(0.018)		(0.019)		(0.027)	
Personal × Woman	0.052**		0.042**		0.071+		0.052**		0.056*		0.049+		0.029		0.050*		0.056*		0.021		0.061**		0.053**		0.055*	
	(0.020)		(0.021)		(0.047)		(0.020)		(0.029)		(0.034)		(0.047)		(0.029)		(0.029)		(0.019)		(0.021)		(0.021)		(0.029)	
(Women's personal coefficient)	0.009		0.004		−0.016		0.009		0.009		0.006		−0.023		0.010		0.017		−0.004		−0.025**		0.010		0.008	
	(0.010)		(0.009)		(0.023)		(0.010)		(0.011)		(0.026)		(0.039)		(0.012)		(0.017)		(0.006)		(0.009)		(0.010)		(0.011)	
Household		−0.022**		−0.022**		−0.029+		−0.022**		−0.037*		−0.014+		‐		−0.032**		−0.012		−0.006		0.011		−0.020**		−0.034*
		(0.009)		(0.009)		(0.018)		(0.009)		(0.018)		(0.010)		‐		(0.013)		(0.011)		(0.006)		(0.011)		(0.009)		(0.018)
Household × Woman		0.004		0.002		−0.001		0.006		0.008		0.004		‐		0.010		0.009		0.002		−0.061		0.003		0.011
		(0.013)		(0.012)		(0.028)		(0.013)		(0.023)		(0.015)		‐		(0.016)		(0.022)		(0.008)		(0.021)		(0.013)		(0.025)
(Women's household coefficient)		−0.018*		−0.020**		−0.030+		−0.016+		−0.028*		−0.010		‐		−0.021**		−0.004		−0.003		−0.015+		−0.017*		−0.023+
		(0.009)		(0.009)		(0.021)		(0.010)		(0.014)		(0.011)		‐		(0.009)		(0.019)		(0.005)		(0.010)		(0.009)		(0.018)
Adjusted r‐squared	0.03872	0.03880	0.02944	0.02966	0.25616	0.25601	0.03914	0.03919	0.04508	0.04529	0.02841	0.02830	0.03001	‐	0.03890	0.03873	0.0385	0.0390	0.01360	0.01338	0.07087	0.07089	0.03872	0.03874	0.04567	0.04577
AIC	55010.12	55006.2	50944.84	50935.02	17348.39	17350.88	53221.61	53219.67	39814.26	39807.02	15155.9	15157.18	9612.032	‐	34651.97	33287.42	22170.15	21879.87	−9962.825	−9910.517	44454.82	44453.96	55009.88	55009.23	38178.35	38175.2
Variance components																										
*Sd(u)*	2.220	2.197	2.325	2.304	3.208	3.208	2.206	2.182	1.757	1.736	3.302	3.289	3.495	‐	2.310	2.120	2.138	2.390	0.312	0.315	1.632	1.606	2.226	2.201	1.747	1.728
*Sd(e)*	0.498	0.498	0.476	0.476	0.573	0.573	0.497	0.497	0.498	0.498	0.499	0.499	0.486	‐	0.502	0.494	0.496	0.507	0.196	0.196	0.478	0.478	0.498	0.498	0.496	0.496
No. of individuals	7780		7780		3889		7520		5475		2305		1525		4861	4819	3182	2973	4173	4117	7487		7780		5244	
No. of data points	43,954		43,954		12,756		42,645		31,755		12,199		8072		27,396	27,033	17,874	16,993	11,431	11,248	38,693		43,954		30,555	

*Note*: Standard errors are clustered for individuals; all models control for level of education (allowed to change by gender), unemployment (allowed to change by gender), socio‐demographic variables (significant coefficients allowed to change by gender) and year (allowed to change by gender). Educational level divides into: primary, vocational, general secondary and academic education; unemployment: has been unemployed in the last year; demographics contain: number of children, age, population density of area of domicile and cohabiting partner. Alternative models excluding education and unemployment lead to the same results.

Models descriptions: Model A: The main model of this paper, as presented in Table A3 and Figure 3. Model B: The dependent variable is sympathy towards PVV and Wilders only. Model C: The dependent variable is sympathy towards FvD and Baudet only. Model D: Only respondents who have an income on average in all time points. Model E: Only respondents who are in a partnership (either married or co‐habiting) in most of the time points. Model F: Only respondents who are not in partnership (either married or co‐habiting) in most of the time points. Model G: Only respondents who are the sole member of their household in most of the time points. Model H: Only respondents whose income trajectory through time is negative. Model I: Only respondents whose income trajectory through time is positive. Model J: Change in dependent variable to voting for RWP parties. A fixed‐effect multilevel linear probability model predicting voting behaviour (see Sipma et al., 2023a for a similar model and a discussion of its advantages against Logit model). Voting behaviour was taken from the closest time point to an election, and income is average from preceding time since last election. Thus we have only four times points, as the number of elections. Model K: Same as Model A, but with extra controls for social trust, satisfaction of government, left‐right political alignment, and attitudes towards immigrants. Model L: Personal and household income are gross income instead of net income. Model M: Only respondents who have an income on average through all time points and who are also in partnership (either married or co‐habiting) in most of the time points.

***p* < .01, **p* < .05, +*p* < .10, one‐tailed tests; Household X Woman is tested using a two‐tailed test.

**TABLE A7:** Standardised coefficients of a fixed‐effects hierarchical linear regression predicting attitudes towards RWP parties, allowing income to change by gender and by who earns more in the household.

Robustness test	Figure 4 Legend	Dependent variable: Sympathies towards RWP parties	Dependent variable: PVV sympathy	Dependent variable: FvD sympathy	Subsample: Gainfully employed only	Subsample: Decreasing income	Subsample: Increasing income	Dependent variable: RWP voting	Control of social trust, satisfaction of government, left‐right political alignment, and attitudes towards immigrants	Independent variable: Gross log income change
Model		(A)	(B)	(C)	(D)	(E)	(F)	(G)	(H)	(I)
Income										
(1) Personal	M|M > W	−0.053*	−0.048+	0.065	−0.053*	−0.060+	−0.010	−0.037	−0.063*	−0.037
		(0.031)	(0.03)	(0.211)	(0.031)	(0.043)	(0.043)	(0.034)	(0.032)	(0.032)
(2) Personal + Personal x Woman	W|M > W	0.014	0.011	−0.001	0.014	0.016	0.021	−0.009+	0.019	0.016
		(0.011)	(0.01)	(0.025)	(0.011)	(0.013)	(0.018)	(0.007)	(0.011)	(0.011)
(3) Personal + Personal x Woman earn more	M|W > M	−0.010	0.001	−0.100+	−0.010	0.062	−0.066+	0.021	−0.022	−0.012
		(0.037)	(0.043)	(0.068)	(0.037)	(0.054)	(0.044)	(0.023)	(0.042)	(0.045)
(4) Personal + Personal x Woman + Personal x Woman earn more + Personal x Woman x Women earn more	W|W > M	−0.079*	−0.103*	−0.173	−0.078*	−0.030	−0.121**	0.026	−0.084*	−0.065+
		(0.043)	(0.059)	(0.330)	(0.043)	(0.055)	(0.035)	(0.026)	(0.042)	(0.041)
(5) Personal x Woman	M|M > W–W|M > W	0.068*	0.059*	−0.066	0.067*	0.076*	0.030	0.028	0.082**	0.053+
		(0.033)	(0.031)	(0.213)	(0.033)	(0.045)	(0.047)	(0.035)	(0.034)	(0.033)
(6) Personal x Woman earn more	M|M > W–M|W > M	0.043	0.049	−0.165	0.043	0.122*	−0.057	0.058+	0.041	0.025
		(0.049)	(0.052)	(0.222)	(0.049)	(0.069)	(0.062)	(0.041)	(0.054)	(0.055)
(7) Personal x Woman earn more + Personal x Woman x Woman earn more	W|M > W–W|W > M	−0.093*	−0.113*	−0.172	−0.092*	−0.046	−0.142**	0.035	−0.103**	−0.081*
		(0.044)	(0.06)	(0.331)	(0.044)	(0.056)	(0.039)	(0.027)	(0.044)	(0.042)
(8) Personal x Woman + Personal x Woman x Woman earn more	M|W > M–W|W > M	−0.069	−0.104+	−0.072	−0.068	−0.092	−0.055	0.005	−0.062	−0.053
		(0.057)	(0.073)	(0.337)	(0.057)	(0.077)	(0.056)	(0.035)	(0.060)	(0.061)
(9) Personal x Woman x Woman earn more	(6) ‐ (7)	−0.136*	−0.162*	−0.007	−0.135*	−0.167	−0.085	−0.023	−0.144*	−0.106
		(0.066)	(0.079)	(0.399)	(0.066)	(0.089)	(0.074)	(0.049)	(0.069)	(0.069)
Adjusted r‐squared		0.04409	0.04416	0.27347	0.03753	0.04363	0.04414	0.01276	0.07516	0.04360
AIC		42691.7	42689.4	12479.4	53273.27	26906.11	17423.19	−7600.22	34726.96	42663.51
Variance components										
*Sd(u)*		2.019	2.121	4.787	0.889	2.549	1.233	0.287	1.449	2.019
*Sd(e)*		0.498	0.475	0.565	0.498	0.500	0.496	0.199	0.478	0.498
No. of individuals		5811	5811	2789	7520	3704	2340	3279	5606	5811
No. of data points		33,965	33,965	9294	42,645	21,286	13,893	9090	29,998	33,965

*Note*: Standard errors are clustered for individuals; all models control for level of education (allowed to change by gender), unemployment (allowed to change by gender), socio‐demographic variables (significant coefficients allowed to change by gender) and year fixed‐effects (allowed to change by gender). Educational level divides into: primary, vocational, general secondary and academic education; unemployment: has been unemployed in the last year; demographics contain: number of children, age, population density of area of domicile and cohabiting partner. Alternative models excluding education and unemployment lead to the same results. Coefficient interpretation: (1) Men, when men earn more; (2) Women, when men earn more; (3) Men, when women earn more; (4) Women, when women earn more; (5) Difference between (1) and (2); (6) Difference between (1) and (3); (7) Difference between (2) and (4); (8) Difference between (3) and (4); (9) Difference between (6) and (7).

Models descriptions: Model A: The main model of this paper, as presented in Table A4 and Figure 4. Model B: The dependent variable is sympathy towards PVV and Wilders only. Model C: The dependent variable is sympathy towards FvD and Baudet only. Model D: Only respondents who have an income on average in all time points. Model E: Only respondents whose income trajectory through time is negative. Model F: Only respondents whose income trajectory through time is positive. Model G: Change in dependent variable to voting for RWP parties. A fixed‐effect multilevel linear probability model predicting voting behaviour (see Sipma et al., 2023a for a similar model and a discussion of its advantages against Logit model). Voting behaviour was taken from the closest time point to an election, and income is average from preceding time since last election. Thus we have only four times points, as the number of elections. Model H: Same as Model A, but with extra controls for social trust, satisfaction of government, left‐right political alignment, and attitudes towards immigrants. Model I: Personal and household income are gross income instead of net income.

***p* < .01, **p* < .05, +*p* < .10, one‐tailed tests; Coefficient 9 is tested using a two‐tailed test.

**TABLE A8:** All standardised coefficients of a fixed‐effects hierarchical linear regression predicting attitudes towards RWP parties, with gender moderating effects, and woman earning more moderation.

Model	(1a)	(2a)	(3a)	(1b)	(2b)
Income					
(1) Personal	0.000	−0.043*	−0.053*		
	(0.009)	(0.018)	(0.031)		
(2) Personal + Personal x Woman		0.052**	0.014		
		(0.020)	(0.011)		
(3) Personal + Personal x Woman earn more			−0.010		
			(0.037)		
(4) Personal + Personal x Woman + Personal x Woman earn more + Personal x Woman x Women earn more			−0.079*		
			(0.043)		
(5) Personal x Woman		0.009	0.068*		
		(0.010)	(0.033)		
(6) Personal x Woman earn more			0.043		
			(0.049)		
(7) Personal x Woman earn more + Personal x Woman x Woman earn more			−0.093*		
			(0.044)		
(8) Personal x Woman + Personal x Woman x Woman earn more			−0.069		
			(0.057)		
(9) Personal x Woman x Woman earn more			−0.136*		
			(0.066)		
(10) Household				−0.020**	−0.022*
				(0.006)	(0.009)
(11) Household x Woman					0.004
					(0.013)
(12) Household + Household x Woman					−0.018*
					(0.009)
Experienced unemployment	0.029*	0.021	−0.020	0.028+	0.023
	(0.017)	(0.027)	(0.034)	(0.017)	(0.027)
Experienced unemployment x woman		0.013	0.043		0.008
		(0.035)	(0.042)		(0.035)
Education level					
Vocational secondary edu.	0.000	−0.015	0.036	−0.001	−0.015
	(0.052)	(0.060)	(0.067)	(0.052)	(0.060)
General secondary edu.	0.037	−0.034	−0.037	0.031	−0.030
	(0.065)	(0.084)	(0.091)	(0.065)	(0.085)
Academic edu.	−0.022	−0.113*	−0.113+	−0.023	−0.117*
	(0.053)	(0.068)	(0.079)	(0.053)	(0.068)
Vocational secondary edu. x Woman		0.024	0.068		0.022
		(0.102)	(0.106)		(0.101)
General secondary edu. x Woman		0.115	0.263*		0.104
		(0.128)	(0.142)		(0.127)
Academic edu. x Woman		0.167+	0.292**		0.168+
		(0.103)	(0.115)		(0.103)
Demographics					
Children	0.015+	0.023+	0.022*	0.017+	0.024+
	(0.009)	(0.012)	(0.013)	(0.009)	(0.012)
Children x woman		−0.014	−0.012		−0.013
		(0.017)	(0.018)		(0.017)
Population density (base = 2500 or more)					
1500–2500	0.008	0.006	0.002	0.008	0.007
	(0.023)	(0.023)	(0.028)	(0.023)	(0.023)
1000–1500	0.003	0.001	−0.002	0.003	0.001
	(0.026)	(0.026)	(0.031)	(0.026)	(0.026)
500–1000	0.005	0.004	0.004	0.006	0.005
	(0.027)	(0.027)	(0.031)	(0.027)	(0.027)
less than 500	−0.021	−0.023	−0.010	−0.020	−0.022
	(0.031)	(0.031)	(0.034)	(0.031)	(0.031)
Age	−0.002	−0.075*	−0.068+	−0.002	−0.074*
	(0.010)	(0.033)	(0.037)	(0.010)	(0.033)
Age x woman		0.084+	0.071+		0.083+
		(0.035)	(0.038)		(0.035)
Partner	−0.007	−0.007	‐	0.002	0.000
	(0.022)	(0.022)	‐	(0.022)	(0.022)
Time fixed‐effects (base = 2010)					
2007	−0.072*	−0.320**	−0.304*	−0.070*	−0.317**
	(0.033)	(0.103)	(0.119)	(0.033)	(0.103)
2008	−0.205**	−0.387**	−0.377**	−0.204**	−0.386**
	(0.023)	(0.069)	(0.079)	(0.023)	(0.069)
2009	−0.190**	−0.316**	−0.318**	−0.190**	−0.316**
	(0.016)	(0.038)	(0.044)	(0.016)	(0.038)
2011	−0.126**	−0.079*	−0.081*	−0.127**	−0.080*
	(0.014)	(0.036)	(0.041)	(0.014)	(0.036)
2012	−0.292**	−0.170+	−0.192*	−0.295**	−0.172+
	(0.024)	(0.068)	(0.079)	(0.024)	(0.068)
2013	−0.225**	−0.048	−0.072	−0.229**	−0.051
	(0.032)	(0.100)	(0.115)	(0.032)	(0.100)
2015	−0.257**	0.091	0.033	−0.262**	0.086
	(0.051)	(0.164)	(0.189)	(0.051)	(0.164)
2016	−0.237**	0.200	0.141	−0.242**	0.194
	(0.061)	(0.197)	(0.226)	(0.060)	(0.196)
2017	−0.178*	0.330	0.256	−0.183**	0.323
	(0.070)	(0.229)	(0.263)	(0.070)	(0.228)
2018	−0.233**	0.342	0.263	−0.239**	0.334
	(0.080)	(0.261)	(0.301)	(0.079)	(0.260)
2019	−0.266**	0.382	0.304	−0.271**	0.373
	(0.089)	(0.295)	(0.339)	(0.089)	(0.293)
2020	−0.371**	0.305	0.201	−0.377**	0.295
	(0.099)	(0.327)	(0.376)	(0.099)	(0.326)
2021	−0.397**	0.364	0.257	−0.402**	0.354
	(0.109)	(0.359)	(0.412)	(0.109)	(0.358)
2007 x Woman		0.306**	0.281*		0.305**
		(0.111)	(0.123)		(0.111)
2008 x Woman		0.238**	0.207*		0.237**
		(0.075)	(0.083)		(0.074)
2009 x Woman		0.185**	0.180**		0.184**
		(0.043)	(0.048)		(0.043)
2011 x Woman		−0.038	−0.024		−0.038
		(0.040)	(0.045)		(0.040)
2012 x Woman		−0.125+	−0.092		−0.125+
		(0.074)	(0.082)		(0.074)
2013 x Woman		−0.177	−0.141		−0.177+
		(0.108)	(0.119)		(0.107)
2015 x Woman		−0.394*	−0.307+		−0.394*
		(0.175)	(0.194)		(0.175)
2016 x Woman		−0.508+	−0.406+		−0.507+
		(0.210)	(0.231)		(0.209)
2017 x Woman		−0.589+	−0.470+		−0.587+
		(0.244)	(0.269)		(0.243)
2018 x Woman		−0.663+	−0.547+		−0.660+
		(0.278)	(0.306)		(0.277)
2019 x Woman		−0.746+	−0.610+		−0.742+
		(0.313)	(0.345)		(0.312)
2020 x Woman		−0.748*	−0.586+		−0.743*
		(0.348)	(0.383)		(0.347)
2021 x Woman		−0.855*	−0.699+		−0.849*
		(0.382)	(0.420)		(0.381)
Average value of fixed‐effects	0.302	1.524*	1.547+	0.281	1.487*
	(0.430)	(0.718)	(0.795)	(0.429)	(0.716)
Adjusted r‐squared	0.03732	0.03871	0.04367	0.03765	0.03880
AIC	55054.0	55010.2	42661.0	55038.9	55006.2
Variance components					
*Sd(u)*	0.891	2.220	2.019	0.890	2.197
*Sd(e)*	0.499	0.498	0.498	0.499	0.498
No. of individuals	7780		5811	7780	
No. of data points	43,954		33,965	43,954	

*Note*: Standard errors are clustered for individuals; Alternative models excluding education and unemployment lead to the same results. Each year in the table fixed‐effects delimits dependent variable collected from December of that year, until March the following year, and independent variables from January to December of hat year.

***p* < 0.01, **p* < 0.05, +*p* < 0.10, one‐tailed tests; for coefficients (9) and (11), demographics and the intercept, two‐tailed tests.
